# Kruppel-like factor 9 may regulate the inflammatory injury of chondrocytes by affecting NF-κB signaling

**DOI:** 10.1186/s13018-025-05974-y

**Published:** 2025-06-18

**Authors:** Haoye Gu, Xingming Han, Yong Ding, Jianhua Deng

**Affiliations:** Department of Joint Surgery, The Sixth People’s Hospital of Nantong, Affiliated Nantong Hospital of Shanghai University, No. 881 Yonghe Road, Chongchuan District, Jiangsu, 226000 China

**Keywords:** KLF9, Osteoarthritis, Inflammatory, CHON-001, Human synovial cells

## Abstract

**Background:**

Kruppel-like factor 9 (KLF9) is involved in the development of osteoarthritis (OA), which is a chronic joint disorder. However, the pathogenesis of OA remains unclear. This study aimed to investigate the relationship between KLF9 and the pathogenesis of OA.

**Methods:**

KLF9 expression in the Gene Expression Omnibus database was analyzed, and the most significantly upregulated and downregulated genes were visualized using a volcano map. Analyses were performed using Gene Ontology and Kyoto Encyclopedia of Genes and Genomes to determine the most significantly changed genes. Interleukin-1 beta (IL-1β) was applied to CHON-001 cells and human synovial cells (HSyCs) to establish an OA in vitro cell model. Real-time quantitative polymerase chain reaction (RT-qPCR) and western blot analysis were conducted to evaluate the expressions of KLF9 and cell death genes. Enzyme-linked immunosorbent assay (ELISA) was conducted to examine IL-1β, interleukin-6 (IL-6), and tumor necrosis factor alpha (TNF-α). IL-1β induced CHON-001 cells; HSyCs were transfected with *KLF9* overexpression (OE); and ELISA was conducted to examine IL-1β, IL-6, and TNF-α. An inhibitor of nuclear factor kappa-light-chain-enhancer of activated B cells (NF-κB) was used, and its effects on CHON-001 cells and HSyC were examined.

**Results:**

*KLF9* was one of the most significantly downregulated genes during OA development. KLF9 was downregulated in IL-1β-treated CHON-001 cells and HSyCs. IL-1β induced the significant upregulation of IL-1β, IL-6, and TNF-α and increased cell death in CHON-001 cells and HSyCs. KLF9 OE partially mitigated the effects of IL-1β and markedly attenuated the IL-1β-induced upregulation of TNF-α and IL-6. IL-1β treatment significantly upregulated B-cell lymphoma 2–associated X protein (Bax) and Caspase-3 and downregulated B-cell lymphoma 2 (Bcl-2) on both messenger ribonucleic acid and protein levels, and KLF9 OE mitigated the effects of IL-1β. IL-1β decreased the levels of type II collagen and aggrecan, whereas KLF9 OE increased the levels of type II collagen and aggrecan. An NF-κB inhibitor could partially abrogate the KLF9-induced effects on Bax, Caspase-3, and Bcl-2. The NF-κB inhibitor also reversed the KLF9 OE–induced increase in the levels of type II collagen and aggrecan.

**Conclusions:**

KLF9 mitigated the IL-1β-induced inflammatory condition via the NF-κB pathway.

**Supplementary Information:**

The online version contains supplementary material available at 10.1186/s13018-025-05974-y.

## Background

Osteoarthritis (OA) is a common global disease, particularly among middle-aged and elderly individuals. Owing to the acceleration of population aging, the incidence rate of OA is increasing year by year. This phenomenon not only leads to an increase in patients suffering from physical pain and dysfunction but also imposes a heavy economic burden on families and society. OA is a degenerative disease that affects many cells and related extracellular matrix (ECM). Chondrocytes and extracellular matrix of chondrocytes together constitute articular cartilage tissue. Any factors that affect the activity of chondrocytes and destroy the metabolic balance of ECM in chondrocytes will lead to the failure of articular cartilage to function normally [23].

Chondrocytes are the main components of articular cartilage and are responsible for synthesizing extracellular matrix (ECM) components and maintaining the integrity of cartilage tissue; therefore, maintaining the synthesis and catabolic homeostasis of chondrocytes is important to normal joint function (Ding, Xiao, & Xu [7], Zheng, Zhang, Sheng, & Mobasheri [31]). However, under the stimulation of various pathological factors, chondrocytes are prone to inflammatory reaction, which leads to a series of cascade injury events, the degeneration and destruction of articular cartilage, and joint dysfunction (H. Yu, Lin, Zhang, Zhang, & Hu [28]). Studies have reported that inflammation can inhibit the synthesis of cartilage matrix proteins [21] and affect the expression of proteolytic enzymes that can degrade the ECM (Haack, Overall, & Auf dem Keller [10], Najafi, Farhood, & Mortezaee [19]). In addition, the expression of cyclooxygenase-2 can be upregulated to increase the levels of the downstream enzymatic reaction product prostaglandin, thus further leading to an imbalance in chondrocyte synthesis and catabolism [8]. Inflammation may lead to alternations in chondrocyte functions possibly owing to the overactivation or inhibition of cellular signaling pathways such as catenin, Smad2/3, and Hedgehog [1, 6, 9, 13]. In addition, inflammation can significantly inhibit the expression of SRY-box transcription factor 9, which is an important transcription factor that is closely related to cartilage formation [22], and upregulate the expression of runt-related transcription factor-2, which is an important transcription factor that promotes chondrocyte differentiation into hypertrophic chondrocytes [11].

In recent years, the Kruppel-like factor (KLF) family, which as an important zinc finger transcription factor, has gradually attracted the attention of researchers. Among them, Kruppel-like factor 9 (KLF9) shows a unique regulatory role in many physiological and pathological processes [29]. In addition to regulating target gene transcription, it can also interact with other transcription factors and coactivators/inhibitors to regulate gene transcription, body metabolism, and oxidative stress [3, 24]. Studies have shown that there is a close relationship between KLF9 and inflammatory reaction and that KLF9 can directly or indirectly participate in the regulation of nuclear factor kappa-light-chain-enhancer of activated B cell (NF-κB) expression of inflammation-related genes [2]. A recent bioinformatic analysis showed that *KLF9* is one of the most differentially expressed genes (DEGs) in OA (E. Yu, Zhang, Xu, Liu, & Yan [27]). However, the role of KLF9 in the inflammatory injury of chondrocytes and its specific molecular mechanism have not been fully explained.

On the basis of the above research background, we speculate that KLF9 may regulate the inflammatory injury process of chondrocytes. This study aimed to clarify the regulatory role and mechanism of KLF9 in chondrocyte inflammatory reaction and provide theoretical basis and potential targets for further understanding the pathogenesis of joint diseases and developing new therapeutic drugs.

## Methods

### Bioinformatics

The levels of KLF9 were determined using the GSE55235 dataset of the Gene Expression Omnibus (GEO) database (https://www.ncbi.nlm.nih.gov/geo/). The data were analyzed using the Xiantao academic analysis tool.

### Cell culture

Human chondrocyte line CHON-001 and primary human synovial fibroblasts cells were used in the current study. Human synovial cells (HSyCs) purchased from Wuhan SUNNcell Biotechnology Co., Ltd. (China). Human synovial cell culture medium (SNPM-H117, SUNNcell Biotechnology Co., Ltd.) was used for the cell culture process. Cells were cultured by 10% fetal bovine serum (Thermofisher, USA) with 1% antibiotics (Thermofisher) at 37 °C under 5% CO_2_.

CHON-001 was purchased from American Type Culture Collection (USA). Dulbecco’s modified Eagle medium (Thermofisher) was used for the cell culture process. Cells were cultured by 10% fetal bovine serum (Thermofisher) with 1% antibiotics (Thermofisher) at 37 °C under 5% CO_2_. Interleukin-1 beta (IL-1β; 2.5, 5, and 10 ng/ml; Thermofisher) was used for treating the cells.

### Transfection

The full-length coding sequence of human KLF9 was obtained from the NCBI website (http://www.ncbi.nlm.nih.gov/). The full-length coding region was PCR-amplified, and the amplified fragment was gel-purified and then ligated into the pcDNA3.1 vector to construct the KLF9 overexpression plasmid. The KLF9 overexpression (OE) plasmid and the OE negative control were purchased from Guangzhou Ribobio Co., Ltd. (China). The PCR primers were as follows: Forward primer: 5′-CGGAATTCATGTCCGCGGCCGCCTACA-3′, Reverse primer: 5′-GCGAAGCTTTCACAAAGCGTTGGCCAGC-3′. These primers contained EcoRI and HindIII restriction sites. Plasmids were delivered to the cells by Lipofectamine 3000 (Thermofisher) on the basis of the information provided by the manufacturers.

### Real-time quantitative polymerase chain reaction (RT-qPCR)

Total ribonucleic acids (RNAs) have been isolated from the cell samples by TRIzol (Invitrogen). Thereafter, complementary deoxyribonucleic acid was reverse transcribed by the kits purchased from Beyotime. RT-qPCR was conducted by ABI 7900 HT system with commercially available kits purchased from Beyotime according to the manufacturer’s instructions. The gene and primer sequences are shown in Table 1, and GAPDH is used as internal reference in KLF9. The relative expression of KLF9 was calculated by 2-^△△CT^ method, and the result was expressed as mean standard deviation.

**Table 1:** Primer sequence

Gene	Sequences	Product size (bp)
KLF9	F: GGGGTTTGGTTTGTGACGTG R: TTTTCCCGAGTCCACTGACG	117
GAPDH	F: GTCAACGGATTTGGTCTGTATT R: AGTCTTCTGGGTGGCAGTGAT	148

### Western blot assay

Firstly, protein in cells or tissues was extracted by RIPI lysis buffer (Gibco, USA) to obtain total protein samples. SDS-PAGE electrophoresis: BCA egg kit (Shanghai Biyuntian Biotechnology Co., Ltd., China) was used to determine the concentration of protein, and SDS-PAGE with a volume fraction of 12% was used for electrophoresis, and the sample quality was 40 µ g per well; After electrophoresis at 80 V for 120 min, the protein was transferred to PVDF membrane at 300 mA and 90 min, and sealed with 5% skim milk powder at room temperature for 1 h;; The PVDF membrane (Millipore, USA) was washed with PBST for 4 times (5 min/ time), and the target protein bands were cut according to the Marker’s position, put into an incubator, and the target protein rabbit primary antibody KLF9 (1: 1000, ABCAM, AB 227920) and bcl 2 (1: 1000, ABCAM, AB 182858) were added respectively. Ab32503), cleaved-caspase 3 (1: 1000, abcam, ab32042) and GAPDH (1: 1000, cell signaling technology, 97166), and then the PVDV membrane was washed with PBST for 3 times (6 min/ time). Accord/ming to the source of antibody species, the goat anti-rabbit or mouse polyclonal antibody (1:10000, China Kangwei Reagent Biotechnology Co., Ltd.) combined with horseradish peroxidase was added, and incubated for 1 h; at room temperature in a shaking table at the speed of 70 ~ 80 rpm. The PVDV membrane was washed with PBST for 3 times (6 min/ time), and the signal of the combination of the secondary antibody and the primary antibody was detected by ECL color development (Millipore, USA). Photographs were taken by chemiluminescence detection system, and the ratio of the optical density of the target band to GAPDH was detected by Image J.

### Statistics

SPSS 23.0 (IBM, USA) was used for the statistical analysis. Data are expressed as mean ± standard deviation. The differences of the two groups were evaluated using Student’s *t*-test, and differences of multiple groups were analyzed using one-way analysis of variance. *P* < 0.05 was considered significant.

## Results

### KLF9 is downregulated in OA

**Fig. 1:**
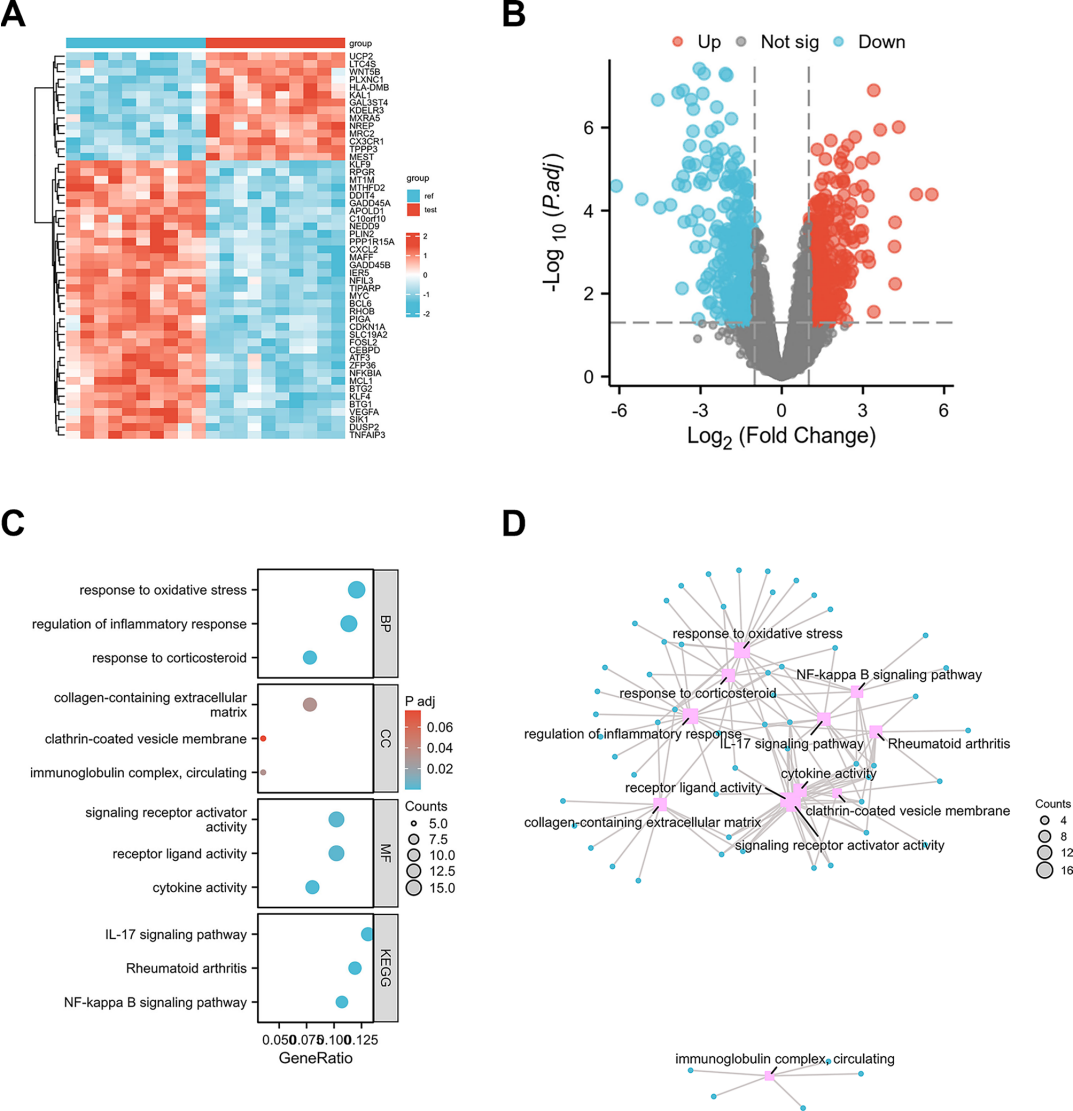
The expression of KLF9 in OA patients and healthy controls. **A**: Heatmap of the GSE55235 chip; **B**: Volcano map of the GSE55235 chip; **C**: GO and KEGG analysis of different expressed genes; **D**: the genes involved process

First, we performed bioinformatic analysis to examine the expression of KLF9 in patients with OA and healthy controls. Figure 1A shows the heatmap of the GSE55235 chip. *KLF9* is one of the most downregulated genes in OA. Moreover, the most significantly upregulated and downregulated genes are presented using a volcano map. As shown in Fig. 1B, genes with LogFC > 1 were calculated; the blue color represents the significantly downregulated genes, whereas the red color represents the significantly upregulated genes. Thereafter, analyses were performed using Gene Ontology and Kyoto Encyclopedia of Genes to evaluate the most significantly changed genes (Fig. 1C). We found that the changed genes were involved in response to corticosteroids, response to oxidative stress, regulation of inflammatory response, collagen-containing ECM, immunoglobulin complex, circulation, clathrin-coated vesicle membrane, cytokine activity, signaling receptor activator activity, receptor ligand activity, rheumatoid arthritis, and NF-κB signaling. We constructed the protein–protein interaction (PPI) network and detected the hub genes in the network. PPI network construction indicated that the changed genes were related to response to oxidative stress, NF-κB signaling pathway, response to corticosteroid, regulation of inflammatory response, rheumatoid arthritis, receptor ligand activity, cytokine activity, etc. (Fig. 1D).

### KLF9 expression is downregulated in CHON-001 cells and HSyCs treated by IL-1β

**Fig. 2:**
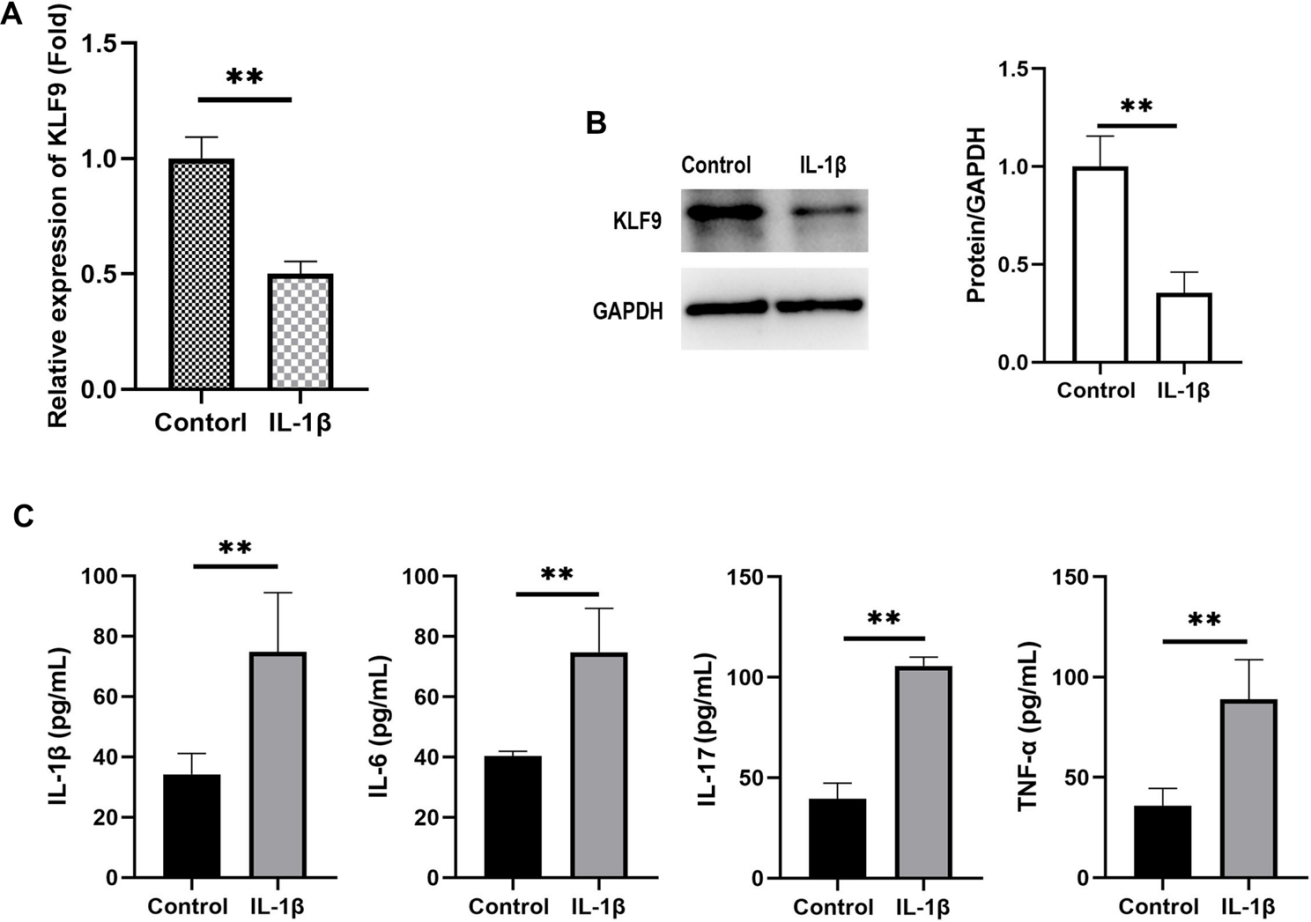
KLF9 expression is down-regulated in CHON-001 cells and HSyC that treated by IL-1β. **A**: The expression of KLF9 in IL-1β treated CHON-001 cells and HSyC was detected through qPCR; **B**: The expression of KLF9 in IL-1β treated CHON-001 cells and HsyC was detected through WB; **C**: ELISA methods were applied for TNF-α as well as IL-6 levels. Number of independent repeated experiments *n* = 3

The levels of KLF9 in in vitro OA models were evaluated. We found that the expression of KLF9 was downregulated in IL-1β-treated CHON-001 cells and HSyCs (Fig. 2A and B). Moreover, enzyme-linked immunosorbent assay (ELISA) was applied for IL-1β, tumor necrosis alpha (TNF-α), and interleukin-6 (IL-6) levels. We found that IL-1β treatment induced the significant upregulation of the above cytokines in CHON-001 cells and HSyCs (Figs. 2C).

### The OE of KLF9 mitigated IL-1β induced inflammatory condition in CHON-001 cells and HSyCs

**Fig. 3:**
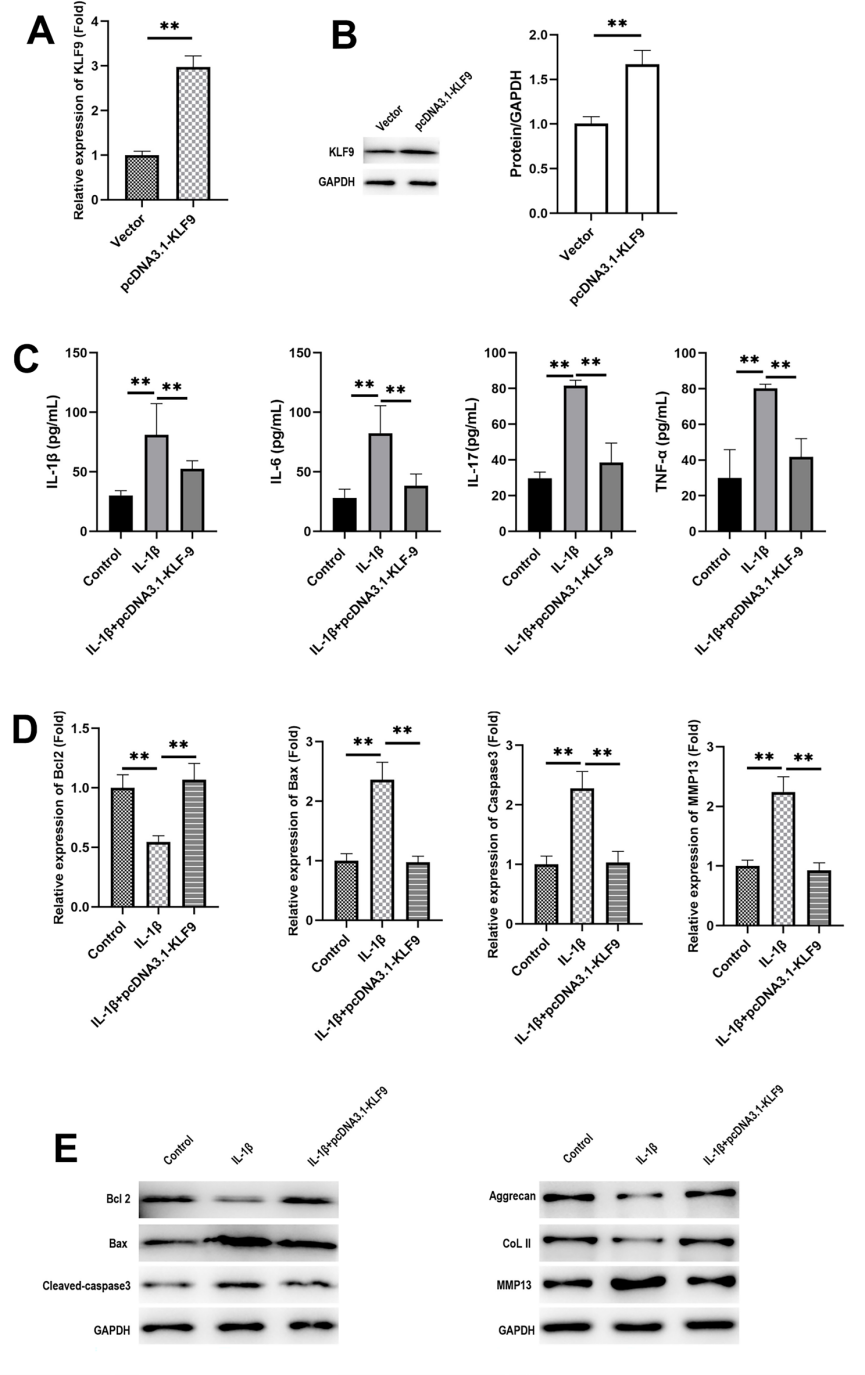
Over-expression of KLF9 alleviated IL-1β included inflammatory condition on CHON-001 cells and HSyC. **A-B**: qPCR and WB detected the transfection efficiency; **C**: ELISA detected the expressions of TNF-α as well as IL-6; **D**: ELISA detected the expressions of Bax, Caspase3 and Bcl-2 on mRNA levels; **E**: WB detected the expressions of Bax, Caspase3, Bcl-2, type II collagen, aggrecan and MMP13 on protein levels. Number of independent repeated experiments *n* = 3

Furthermore, IL-1β induced CHON-001 cells and HSyCs were treated with KLF9 OE, and Fig. 3A and B showed the transfection efficiency. The ELISA results showed that KLF9 OE markedly decreased the IL-1β-induced increased expressions of IL-1β, TNF-α, and IL-6 (Fig. 3C). IL-1β induced the significant upregulation of B-cell lymphoma 2–associated X protein (Bax) and Caspase-3 and the downregulation of B-cell lymphoma 2 (Bcl-2) on both messenger RNA (mRNA) and protein levels, whereas KLF9 OE mitigated the effects of IL-1β (Fig. 3D). Moreover, IL-1β also decreased the expression of type II collagen and aggrecan, whereas KLF9 OE increased the levels of type II collagen and aggrecan in IL-1β-treated CHON-001 cells and HSyCs (Fig. 3E).

### KLF9 mitigated the IL-1β-induced inflammatory condition in CHON-001 cells and HSyCs via the NF-κB pathway

**Fig. 4:**
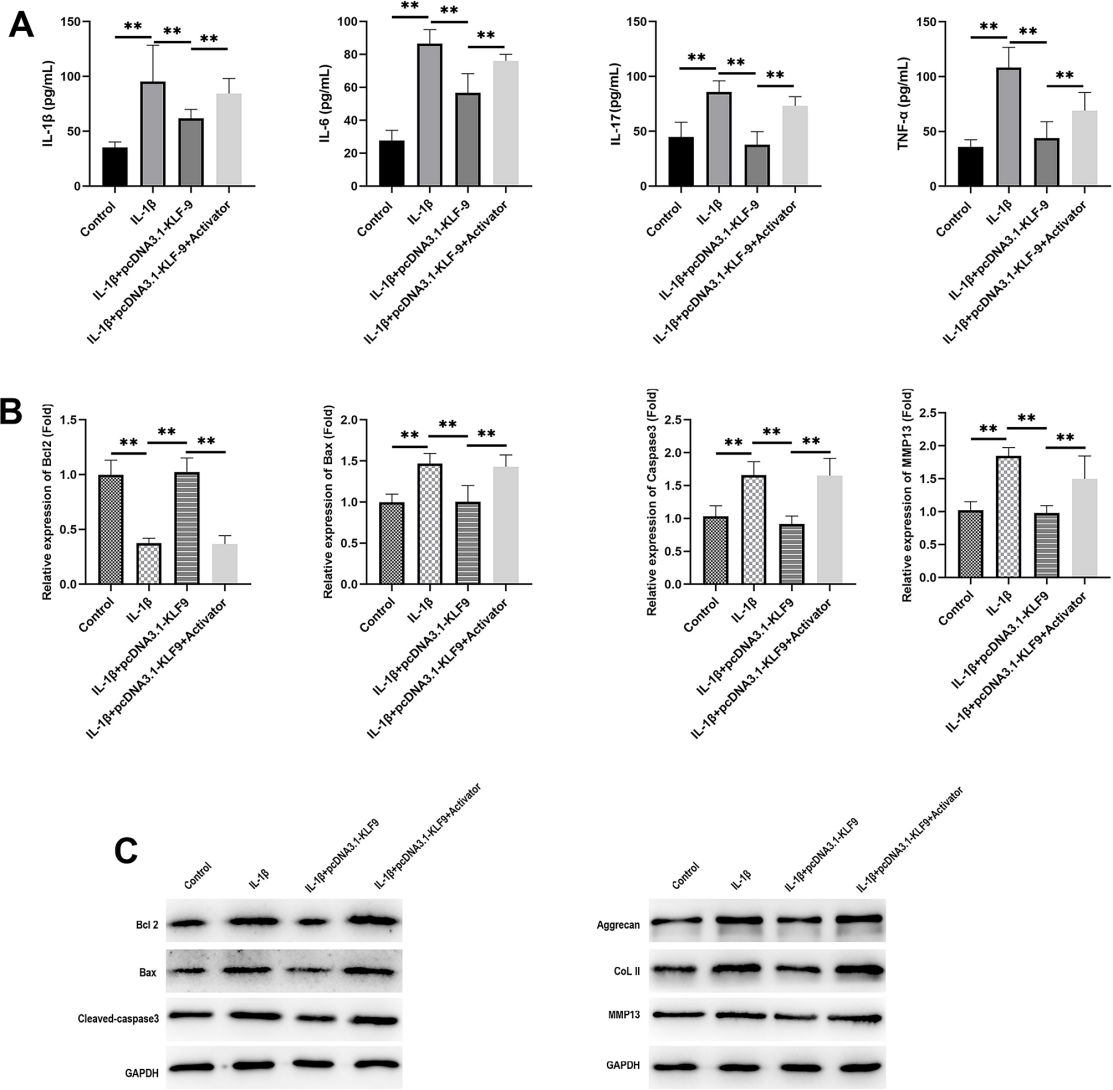
NF-kB inhibitor abrogated the effects of KLF9 OE on CHON-001 cells and HSyC that treated by IL-1β. **A**: ELISA detected IL-1β, IL-6 and TNF-α expressions; **B**: qPCR detect the mRNA expression of Bcl2, Bax, Caspase3 and MMP13; **C** WB detected the expressions of Bax, Caspase3, Bcl-2, type II collagen, aggrecan and MMP13 on protein levels. Number of independent repeated experiments *n* = 3

Finally, the NF-κB inhibitor was used, and Fig. 4A shows that the NF-κB inhibitor partially abrogated the effects of KLF9 OE on IL-1β, TNF-α, and IL-6 expressions in IL-1β-treated CHON-001 cells and HSyCs. Thereafter, the NF-κB inhibitor partially abrogated the KLF9-induced effects on Bax, Caspase-3, and Bcl-2 on both mRNA and protein levels (Fig. 4B). Furthermore, NF-κB inhibitor also reversed the effects of KLF9 OE on type II collagen as wells aggrecan expressions (Fig. 4C).

## Discussion

OA is a common and complicated chronic joint disease, and its pathological mechanism involves gradual articular cartilage degeneration, inflammatory reaction, cytokine network imbalance, and cell death pathway activation. We found that the expression of KLF9 was downregulated in IL-1β-treated CHON-001 cells and HSyCs. At the same time, KLF9 can also mitigate the IL-1β-induced inflammatory state. Furthermore, we found that KLF9 expression was downregulated in IL-1β-treated CHON-001 cells and HSyCs and that KLF9 could alleviate the IL-1β-induced inflammatory condition.

KLF9 encodes the gene in human chromosome 9q13 and was first cloned by Imataka in 1992. This transcription factor has a typical structure of KLF family members: the N-terminus contains abundant Asp/Glu acidic amino acid residues, and the C-terminus contains three tandem Cys2/His2 zinc finger structures [12]; Li, Zhang, Zhang, Liu, & Liu [14]. In addition to regulating the target gene transcription, it can also interact with other transcription factors and coactivators/inhibitors to regulate gene transcription, body metabolism, and oxidative stress [3, 24]. A recent bioinformatic analysis showed that KLF9 is one of the most DEGs in OA [27]. In the current study, we conducted bioinformatics analysis by using the GEO database and found that the expression of KLF9 in OA samples was significantly downregulated; this finding was consistent with previous studies on KLF9 in other inflammatory diseases and degenerative diseases. This result suggests that KLF9 may play a protective role in the development of OA as a key regulatory factor. To further verify this hypothesis, we used IL-1β to treat CHON-001 cells and HSyCs to construct an in vitro cell model of OA. IL-1β is a known pro-inflammatory cytokine that plays a central role in the pathological process of OA. IL-1β treatment further reduced the expression of KLF9 in CHON-001 cells and HSyCs and significantly increased the inflammatory factors IL-6 and TNF-α. This finding echoes the research results of Zhang et al., who found that IL-1β can promote the expression of inflammatory factors by activating the NF-κB signaling pathway [30].

To further explore the role of KLF9 in OA, we conducted an OE experiment of KLF9. The results showed that KLF9 OE could partially reverse the inflammatory reaction and cell death induced by IL-1β. Specifically, KLF9 OE significantly reduced the expression of TNF-α and IL-6, thus indicating that KLF9 might alleviate the inflammatory state by inhibiting the expression of these inflammatory factors. In addition, KLF9 OE also reduced the effect of IL-1β on the apoptosis-related genes *BAX*, Caspase-3, and *BCL2*. These results are consistent with the research results of Elife et al., who found that KLF9 can regulate the mitochondrial apoptosis pathway to protect cells from inflammatory damage [25].

The degradation of the ECM was correlated with the development of OA [17]. Chondrocytes can produce the proteoglycans, and they will be degraded by matrix metalloproteinases (MMPs) (Mehana, Khafaga, & El-Blehi [16]). Collectively, MMPs were also important mediators of OA [4]; Mixon, Savage, Bahar-Moni, Adouni, & Faisal [18]). In the current study, IL-1β markedly decreased the protein expressions of type II collagen and aggrecan, whereas KLF9 OE could upregulate type II collagen and aggrecan expressions. These results suggested that KLF9 may alleviate the IL-1β-induced degradation of ECM. Studies have also mentioned that KLF9, as a transcription factor, has the property of binding to the promoter region of a specific gene and regulating transcription activity [26]. This indicates that KLF9 upregulates the expression of type II collagen and aggrecan, which may be environment dependent in a chondrocyte environment.

OA has been reported to be associated with NF-κB signaling and has received increasing research attention (Choi, Jo, Park, Kang, & Park [5]). NF-κB is a pleiotropic nuclear transcription factor that exists in the cytoplasm of almost all types of cells, usually in an inactive form. NF-κB plays a central role in the response of cells to stimuli such as pressure, cytokines, free radicals, ultraviolet rays, and antigens; participates in inflammatory response and immune response; and regulates cell apoptosis and stress response [20]. When cells are stimulated by external inflammation, the NF-κB signaling pathway is activated, thus promoting gene transcription and the expression of various inflammatory mediators, which trigger and amplify inflammatory reactions (X. Zhang et al., [30]. In chondrocytes, an abnormally activated NF-κB pathway can induce a large number of pro-inflammatory cytokines such as IL-1β and TNF-α, which further act on chondrocytes, thus causing pathological changes such as metabolic imbalance, matrix degradation, and apoptosis and accelerating the degradation process of articular cartilage [15]. To verify whether KLF9 works through NF-κB pathway, we used an NF-κB inhibitor and observed its effect on CHON-001 cells and HSyCs. The results showed that an NF-κB inhibitor could partially eliminate the effects of KLF9 on Bax, Caspase-3, and Bcl-2 and reverse the increase in type II collagen and aggrecan induced by KLF9 OE. This discovery further supports the view that KLF9 exerts its chondroprotective effect by regulating the NF-κB signaling pathway.

## Conclusions

In summary, this study revealed the new function of KLF9 in OA, namely, it can reduce IL-1β-induced inflammation and chondrocyte death by regulating the NF-κB signaling pathway. These findings not only deepen our understanding of the pathological mechanism of OA but also provide a theoretical basis for developing new therapeutic strategies for KLF9 or its downstream signal molecules. However, we only performed cell experiments, which could not accurately reflect the function and regulation mechanism of KLF9 under physiological conditions. Further studies need to conducted to verify in vivo models or clinical studies to ensure their effectiveness and safety in practical applications.

## Electronic supplementary material

Below is the link to the electronic supplementary material.

**Supplementary Material 1**: **Figure 1**: The expression of KLF9 in OA patients and healthy controls. A: Heatmap of the GSE55235 chip; B: Volcano map of the GSE55235 chip; C: GO and KEGG analysis of different expressed genes; D: the genes involved process.

**Supplementary Material 2**: **Figure 2**: KLF9 expression is down-regulated in CHON-001 cells and HSyC that treated by IL-1β. A: The expression of KLF9 in IL-1β treated CHON-001 cells and HSyC was detected through qPCR; B: The expression of KLF9 in IL-1β treated CHON-001 cells and HsyC was detected through WB; C: ELISA methods were applied for TNF-α as well as IL-6 levels.

**Supplementary Material 3**: **Figure 3**: Over-expression of KLF9 alleviated IL-1β included inflammatory condition on CHON-001 cells and HSyC. A-B: qPCR and WB detected the transfection efficiency; C: ELISA detected the expressions of TNF-α as well as IL-6; D: ELISA detected the expressions of Bax, Caspase3 and Bcl-2 on mRNA levels; E: WB detected the expressions of Bax, Caspase3, Bcl-2, type II collagen, aggrecan and MMP13 on protein levels.

**Supplementary Material 4**: **Figure 4**: NF-kB inhibitor abrogated the effects of KLF9 OE on CHON-001 cells and HSyC that treated by IL-1β. A: ELISA detected IL-1β, IL-6 and TNF-α expressions; B: qPCR detect the mRNA expression of Bcl2, Bax, Caspase3 and MMP13; C: WB detected the expressions of Bax, Caspase3, Bcl-2, type II collagen, aggrecan and MMP13 on protein levels.

## Data Availability

Data presented in this study are available on reasonable request from the corresponding author.
