# Supplementary figures and images for: Kruppel-like factor 9 may regulate the inflammatory injury of chondrocytes by affecting NF-κB signaling
Source: J Orthop Surg Res. 2025 Jun 18;20:599. doi: 10.1186/s13018-025-05974-y (PMC12175337; doi:10.1186/s13018-025-05974-y)

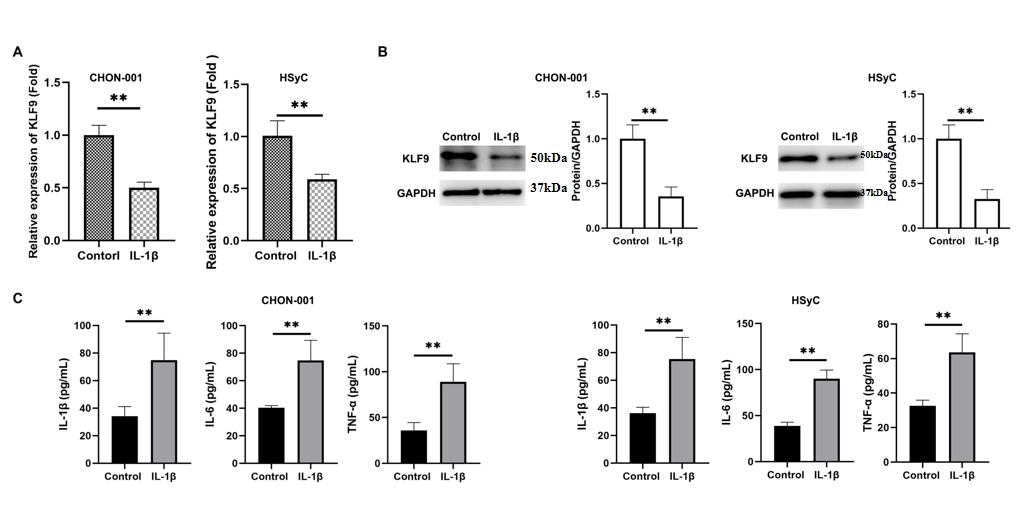

Supplement: Supplementary file 1 — Supplementary Material 1: Figure 1: The expression of KLF9 in OA patients and healthy controls. A: Heatmap of the GSE55235 chip; B: Volcano map of the GSE55235 chip; C: GO and KEGG analysis of different expressed genes; D: the genes involved process. [file 13018_2025_5974_MOESM1_ESM.png]

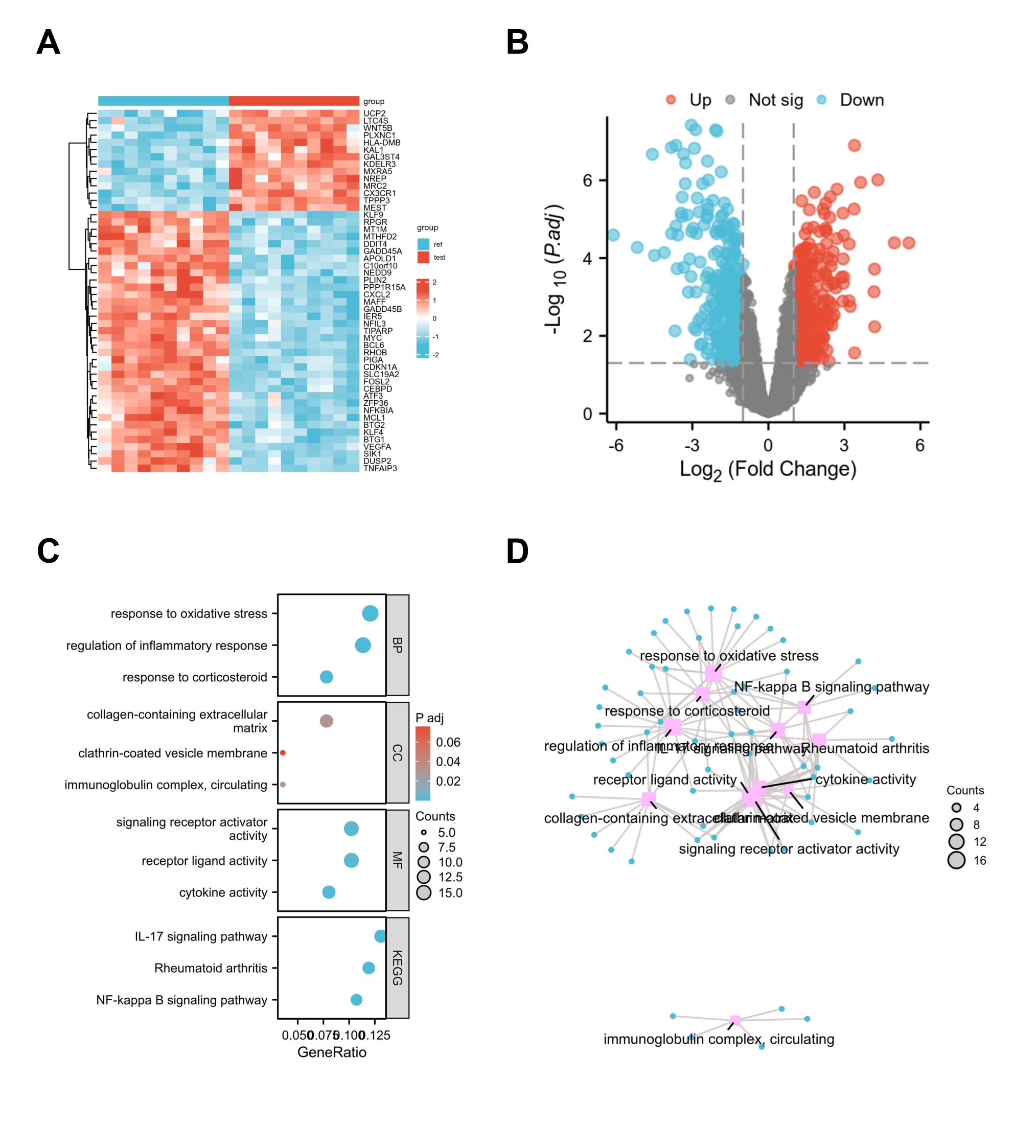

Supplement: Supplementary file 2 — Supplementary Material 2: Figure 2: KLF9 expression is down-regulated in CHON-001 cells and HSyC that treated by IL-1β. A: The expression of KLF9 in IL-1β treated CHON-001 cells and HSyC was detected through qPCR; B: The expression of KLF9 in IL-1β treated CHON-001 cells and HsyC was detected through WB; C: ELISA methods were applied for TNF-α as well as IL-6 levels. [file 13018_2025_5974_MOESM2_ESM.tif]

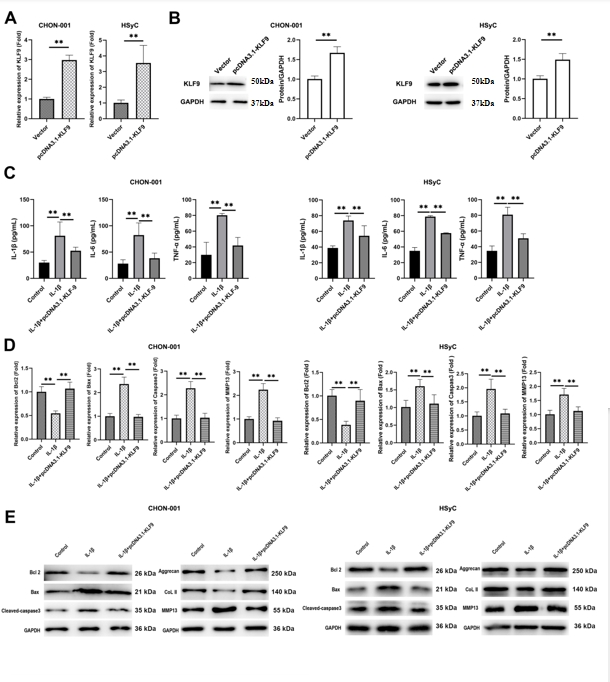

Supplement: Supplementary file 3 — Supplementary Material 3: Figure 3: Over-expression of KLF9 alleviated IL-1β included inflammatory condition on CHON-001 cells and HSyC. A-B: qPCR and WB detected the transfection efficiency; C: ELISA detected the expressions of TNF-α as well as IL-6; D: ELISA detected the expressions of Bax, Caspase3 and Bcl-2 on mRNA levels; E: WB detected the expressions of Bax, Caspase3, Bcl-2, type II collagen, aggrecan and MMP13 on protein levels. [file 13018_2025_5974_MOESM3_ESM.png]

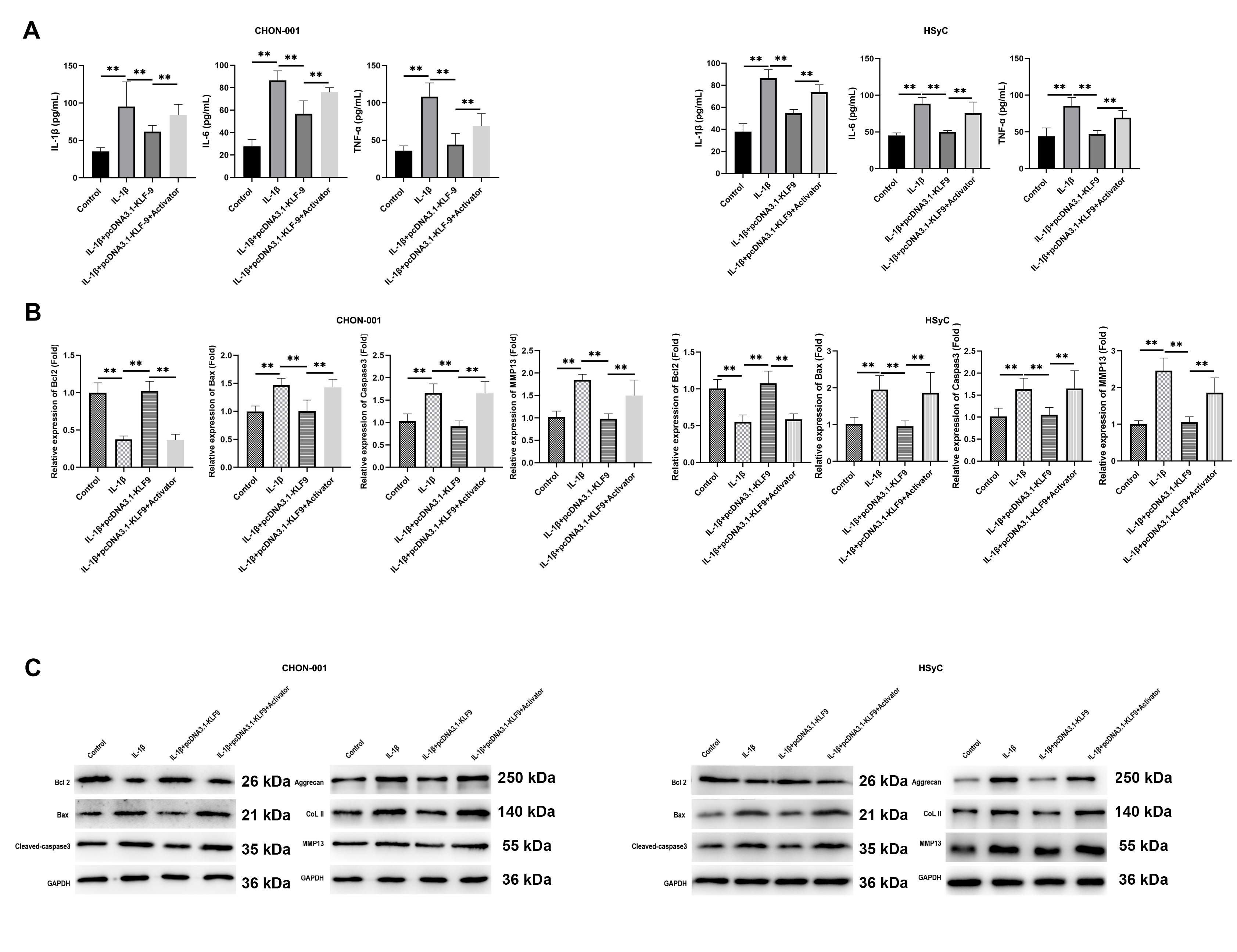

Supplement: Supplementary file 4 — Supplementary Material 4: Figure 4: NF-kB inhibitor abrogated the effects of KLF9 OE on CHON-001 cells and HSyC that treated by IL-1β. A: ELISA detected IL-1β, IL-6 and TNF-α expressions; B: qPCR detect the mRNA expression of Bcl2, Bax, Caspase3 and MMP13; C: WB detected the expressions of Bax, Caspase3, Bcl-2, type II collagen, aggrecan and MMP13 on protein levels. [file 13018_2025_5974_MOESM4_ESM.tif]
